# Data Acquisition Based on Stable Matching of Bipartite Graph in Cooperative Vehicle–Infrastructure Systems †

**DOI:** 10.3390/s17061327

**Published:** 2017-06-08

**Authors:** Xiaolan Tang, Donghui Hong, Wenlong Chen

**Affiliations:** College of Information Engineering, Capital Normal University, Beijing 100048, China; tangxl@cnu.edu.cn (X.T.); fromhdh@163.com (D.H.)

**Keywords:** vehicular networks, data acquisition, stable matching, bipartite graph, content replication

## Abstract

Existing studies on data acquisition in vehicular networks often take the mobile vehicular nodes as data carriers. However, their autonomous movements, limited resources and security risks impact the quality of services. In this article, we propose a data acquisition model using stable matching of bipartite graph in cooperative vehicle-infrastructure systems, namely, DAS. Contents are distributed to roadside units, while vehicular nodes support supplementary storage. The original distribution problem is formulated as a stable matching problem of bipartite graph, where the data and the storage cells compose two sides of vertices. Regarding the factors relevant with the access ratio and delay, the preference rankings for contents and roadside units are calculated, respectively. With a multi-replica preprocessing algorithm to handle the potential one-to-many mapping, the matching problem is addressed in polynomial time. In addition, vehicular nodes carry and forward assistant contents to deliver the failed packets because of bandwidth competition. Furthermore, an incentive strategy is put forward to boost the vehicle cooperation and to achieve a fair bandwidth allocation at roadside units. Experiments show that DAS achieves a high access ratio and a small storage cost with an acceptable delay.

## 1. Introduction

A cooperative vehicle-infrastructure system is a hybrid vehicular network, composed of mobile vehicles and fixed roadside units. The vehicular nodes are equipped with GPS modules, various sensors (e.g., the speed sensor, the road condition sensor, and the inter-vehicle distance sensor), and the wireless communication interfaces to support the dedicated short-range communications (DSRC) [1]. In addition, the roadside units have more powerful computing capability, communication capacity and storage capacity, and often take the role of the internet access points because of their robust and reliable connections via wired links or long-distance high-bandwidth wireless links [2,3]. As a key technology in intelligent transportation systems, vehicular networks work for the safe and comfortable driving, the traffic monitoring, the multimedia sharing and so forth, with the provision of vehicle-to-vehicle (V2V) communications and vehicle-to-roadside-unit (V2U) communications.

Data acquisition in vehicular networks is an important and challenging task due to the various user requirements and the complex network environments. There are lots of practical issues to overcome, such as how to utilize cloud resources and information-centric networking to improve multimedia delivery [4,5], how to circumvent driver distraction when manipulating built-in applications [6], and how to design a smart car with ubiquitous computing and intuitive human-computer interaction [7]. Additionally, smart applications like E-health based on the internet of vehicles are promising future domains [8].

For the content queries sent by the vehicular users, if the mobile vehicular nodes download the contents from the internet directly, a drainage of network resources and severe access congestion are inexorable consequences. Thus, recent research focuses on how to replicate and distribute the contents to vehicular nodes or roadside units in vehicular networks [9]. Although a vast majority of existing studies do research on the content distribution at vehicular nodes, more and more evidence shows that the opportunistic contact chances and the short communication durations in companies with the security risks of autonomous mobile nodes [10] severely restrict the performance enhancements. Therefore, in a cooperative vehicle-infrastructure system, the roadside units with rich resources, such as the storage spaces and the communication bandwidths, may improve the data distribution and provide better data services. In our previous work [11], we briefly introduced a content replication scheme using roadside units as content carriers. However, with respect to the cooperation of vehicles and roadside units, there is still room for improvement.

**Figure 1:**
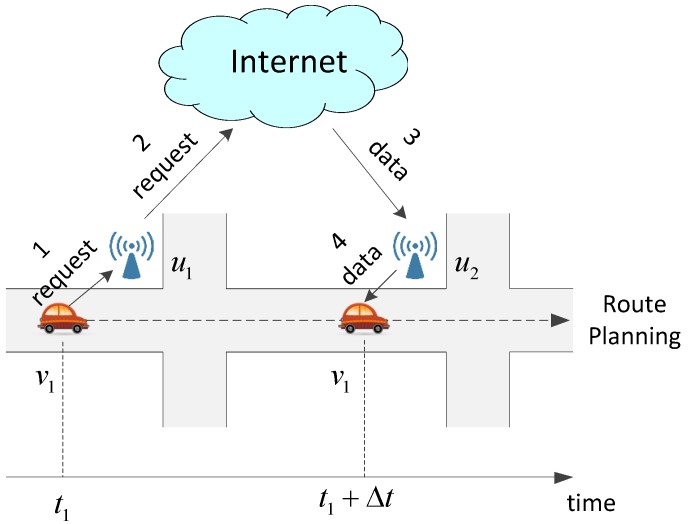
A sketch map of a content request and acquisition process.

This article puts forward an efficient data acquisition model using stable matching of bipartite graph, namely DAS, aiming for a polynomial-time solution which combines the metrics, i.e., the access ratio and the access delay, by problem formulation. A sketch map of a content request and acquisition process in our model is displayed in Figure 1. A roadside unit $u_{1}$ obtains a request from a vehicle $v_{1}$ at time $t_{1}$, and forwards this request to the internet. Then, the online control center computes a content distribution solution and allocates the data to another roadside unit $u_{2}$ accordingly. Thereafter, $u_{2}$ responds to $v_{1}$ at a later time $t_{1}+\Delta t$. In this process, the content distribution solution is a key part affecting the performance of data access, which is determined by the network states, i.e., the requests from vehicles, the storage of roadside units and the vehicle route planning. In this article, through taking the contents as the left vertices and the roadside units as the right vertices in a bipartite graph, and then calculating a preference ranking or a priority ranking for each vertex over vertices in the other side, a stable data distribution solution is obtained, which considers the access ratio metric and the access latency metric together.

Specifically, our main contributions include: (1) the data distribution problem in vehicular networks, as an NP-complete problem, is transformed into a stable matching problem of bipartite graph, which can be solved in polynomial time; (2) since the preference ranking for each content and the priority ranking for each roadside unit are determined by the access ratio and the access latency, DAS is a comprehensive solution for the both criteria; (3) the application scope is enlarged in that a multi-replica content preprocessing algorithm handles the possible one-to-many mapping; and (4) the supplementary storage at vehicular nodes with an incentive strategy overcomes the transmission failures at roadside units due to bandwidth competition to some extent.

The reminder of this article is organized as follows. The related work on data acquisition in vehicular networks is surveyed in Section 2. After analysing the network scenario and presenting the problem statement in Section 3, we introduce the DAS model in detail in Section 4 and Section 5. Section 6 shows the experimental results with relevant analysis, and, eventually, Section 7 concludes this article.

## 2. Related Work

Data acquisition models are usually discussed together with the routing protocols in vehicular networks. In recent years, node cooperation is utilized in data acquisition for vehicular networks. In [12], several autonomous mobile nodes compose a content replication group. Each node replicates a subset of all the contents from the server respectively and shares them with other nodes in its group at a small cost. Thus, the overall access ratio is improved. In [13], based on peer-to-peer (P2P) communication principles, Caviglione and Cervellera construct an optimized framework to manage content replication and dissemination through a mobile vehicular network. Their solution overcomes the bandwidth restriction problem in inter-vehicle content distribution and regards a vehicular flow as a virtual backbone in the scenarios without fixed infrastructures. In [14], Kapadia et al. focus on a static content replication under the random walk mobility model. They construct an optimal formula with constraints to find a good distribution schema, and analyse the influences of relevant network parameters, such as the total system storage, the data item size and the vehicle trip duration. For the online video applications, Kumar and Kim, in [15], propose a probabilistic trust-aware data replication strategy. It uses a data replication tree to represent the transmission process of a content from its source to its destination, and defines a replica cost function and a trust calculation metric for replica location and data replication with read and update operations.

The data acquisition models mostly use mobile vehicular nodes to carry contents. Specifically, according to the requests and the mobility features of vehicles, the number of content replicas and their appropriate carriers are calculated. Thereafter, these carriers respond to the requests via V2V communications when encountering those request senders during their travel time.

Considering the deficiency of existing data replication schemes which take a complete content as the replication unit, Zhuo et al. do research on packet-level data replication in [16]. They explore the contact-duration-aware data replication problem in theory and give a centralized solution to better utilize the limited storage and communication chances. In addition, a distributed replication algorithm is designed with a low computing complexity. Experiments based on synthetic and realistic trajectories confirm the close-to-optimal performance of this proposal. Regarding the content replicas as open resources, La et al. put forward a data distribution scheme using local measurements only, which well approximates an optimal solution and remains robust against network and demand dynamics [17]. Nevertheless, this scheme applies to mobile networks with low velocity, since it is based on multi-hop data transfers among mobile nodes. In [18], Wu et al. model the relationships among the number of replicas of a packet, the replication limit and the queue length of vehicular nodes. Then, a distributed capacity-constrained replication scheme is proposed to dictate how each vehicle adaptively determines its replication strategy subject to the current network capacity. Experiments based on real vehicle GPS traces show a high access ratio. In [19,20], two origin-destination-based content replication schemes, i.e., ODCRep and GO-DCR, select appropriate nodes to replicate contents based on the vehicles’ origin and destination points. Specifically, ODCRep controls the number of replicas in the scenario via efficient algorithms, and hence achieves high coverage and saves resources, while GO-DCR, as a geo-localized solution, increases content availability and reduces delivery cost.

Regarding the significance of roadside infrastructure on the data delivery in cooperative vehicle-infrastructure systems, some studies analyze the features of V2U communications and optimize the deployment of roadside units [21,22,23]. Silva et al. use the migration ratios between adjacent urban cells to obtain α locations for roadside units, which maximize the number of vehicles having at least one vehicle-to-infrastructure contact opportunity [24]. The results show that full knowledge of vehicle trajectories is not necessary for a close-to-optimal deployment. In [25], content delivery networks are developed in the context of vehicular networks to measure the content delivery, and the deployment of roadside units is improved to support the dissemination of various contents requiring different levels of performance. In [26], aiming for ρ percent of vehicles encountering roadside units in time intervals shorter than τ seconds, the Gamma Deployment metric is formalized by developing an Integer Linear Programming formulation, and a heuristic solution is proposed for large instances, which shows small deviations for short inter-contact time guarantees. To achieve short download delays, Luan et al., in [27], replicate contents to lightweight low-cost roadside buffers based on an analytical model of download delay of files. These works focus on the deployment of roadside units, but how to better the utilization of existing infrastructure is still an open issue.

Since the above approaches utilize the mobile vehicles other than the roadside infrastructures to replicate and carry contents, their performances are greatly constrained. Therefore, the key insight of this article is to explore the rich resources of the fixed roadside units to improve the efficiency of data acquisition in cooperative vehicle-infrastructure systems.

## 3. Network Scenario and Problem Statement

We investigate a vehicular scenario including static roadside units with the provision of internet access, and mobile vehicular nodes equipped with devices offering V2V and V2U communication capabilities (e.g., IEEE 802.11p).

A content request and acquisition process starts with a vehicular node sending out a content query to a meeting roadside unit. The roadside unit gathers the queries during a segment of time, named a storage cycle and denoted by τ, and then passes these queries to an online control center. The control center determines a data distribution solution based on a comprehensive analysis of the queries from vehicular nodes, the storage of roadside units and the vehicle routing plans. After the contents are distributed to those roadside units which may encounter the request senders in the next storage cycle τ+1, the requests could be satisfied by the units via V2U communications.

Note that, in our scheme, the data distribution is executed at the beginning of a storage cycle, and then the contents will be carried by relevant roadside units during the whole cycle. When the next storage cycle starts, the new distribution determines whether the already existing contents should be deleted or not. For example, at the start of τ+1, for content carried by a roadside unit in τ, if it is not allocated to this unit currently, it is erased from the storage spaces; otherwise, it remains there during τ+1.

Although the network updating after a storage cycle takes some time, it may be short and acceptable for three reasons. First, the data distribution solution is calculated by the control center with a high computing and communication capability, rather than the resource-restricted vehicular nodes and roadside units. Additionally, the updating time of a roadside unit is only used for data receiving from the control center. In other words, except this time, the roadside units are always serving for vehicular nodes’ data queries. Moreover, dividing a large scenario into several small areas as well as a relatively short storage cycle is helpful to reduce the amount of data and hence shorten the updating time.

For a clear presentation, the primary symbols that will be used throughout this article are listed in Table 1 with some comments below.

In the content request matrix *F*, if a vehicular node $v_{i}$ requests content $c_{k}$ in τ, then $f_{ik}=1$; otherwise, $f_{ik}=0$. In the encountering matrix *G*, if a vehicular node $v_{i}$ will encounter a roadside unit $u_{j}$ in τ+1 according to its routing plan, then $g_{ij}=1$; otherwise, $g_{ij}=0$. In addition, in the encountering latency matrix *H*, if a roadside unit $u_{j}$ is the *x*th one which will be met by a vehicular node $v_{i}$ in τ+1, then $h_{ij}=x$ (0<x≤|U|); otherwise, $h_{ij}=0$. Content C is originally hosted on a server connected with the internet, which can be downloaded through the broadband access at U. We assume the valid duration of a data query equals τ; each content has the same size *s*; the storage capacity of a roadside unit is an integer multiple of *s*. The storage space for a content is known as a storage cell, so the number of storage cells in a roadside unit is an integer, i.e., $w\left(j\right)\in N^{+}$.

From the content request and acquisition process, it is observed that the data replication at roadside units affects the number of satisfied requests and hence the access ratio, and influences the access delay considering the waiting time for encounters between vehicular nodes and roadside units. Taking the maximum access ratio as an objective, the problem of data distribution to roadside units is similar to a 0–1 knapsack problem with the number of satisfied requests as the value and the size of content as the weight. Since several replicas of the same content at different roadside units may respond to the same request, the values of these replicas are not independent. Since the 0–1 knapsack problem is NP-complete, the data distribution problem, which is more complicated than that, is also an NP-complete problem. Therefore, in this paper, we attempt to find a solution in polynomial time, whose ultimate goal is to achieve a high access ratio with acceptable access latency.

In order to clearly define the main metrics including the access ratio and delay, we denote the content distribution results by $D={\left(d_{kj}\right)}_{k\in C,j\in U}$. If content $c_{k}$ is distributed to a roadside unit $u_{j}$, then $d_{kj}=1$; otherwise, $d_{kj}=0$. Using the symbols in Table 1, we get that the access ratio is computed by

(1) $$Z_{AR}=\frac{\sum_{i\in V}\sum_{k\in C}f_{ik}\cdot sgn\left(\sum_{j\in U}g_{ij}d_{kj}\right)}{\sum_{i\in V}\sum_{k\in C}f_{ik}},$$

where sgn() is a sign function, and specifically sgn(x)=1 when x>0, and sgn(x)=0 when x=0. In addition, the average number of roadside units which vehicles encounter before getting data, indicating the access delay, is computed by

(2) $$Z_{AD}=\frac{\sum_{i\in V}\sum_{k\in C}f_{ik}\cdot \min_{j\in U}\left\{h_{ij}|g_{ij}d_{kj}>0\right\}}{\sum_{i\in V}\sum_{k\in C}f_{ik}\cdot sgn\left(\sum_{j\in U}g_{ij}d_{kj}\right)}.$$

In our scheme, we calculate the preference rankings for contents and roadside units based on the access ratio and the delivery delay, aiming to balance these metrics.

Although DAS is based on the aforementioned assumptions, its application scope could be enlarged via simple adjustments—(1) Subject to the popularity, the different contents may have different storage times. For example, a popular video may be stored longer than an unpopular one because more vehicular users would like to download it. Since the content distribution is carried out at each storage cycle, a popular content may be allocated to the same roadside unit in several consecutive storage cycles when its queries continue to exist. In other words, popular content may be stored for more than one storage cycle in DAS; (2) Apparently, different contents may have different sizes. For instance, a video typically has a much larger size than an image. To deal with the variability in size, a preliminary step is used to make the content size equal. Specifically, a content with size b×s ($b\in N^{+}$) is separated into *b* sub-items, each of which has size *s*.

How to distribute the contents C to roadside units U can be regarded as a bipartite graph matching problem with C and U as the left and right vertices, respectively. For a high access ratio and a short access delay, how to solve the bipartite graph matching problem with the prior knowledge of the relationships among C, V and U is our primary motivation.

## 4. Data Acquisition via V2U Communications

### 4.1. Data Distribution at Roadside Units Based on Stable Matching of Bipartite Graph

Stable matching is a specific approach in bipartite graph matching theory. It requires that each vertex on one side has a strict preference ranking over the vertices of the other side. A matching between the left vertices and the right vertices is stable if there is no left–right pair who both strictly prefer each other to their current partners. The concept of stability captures fairness conditions for market participants and has had enormous influence on the design of real world matching markets [28]. In stable matching theory, take matching students and colleges as an example. The goal is to design a self-reinforcing admissions process given a set of preferences among colleges and students. Student *x* and college *y* are unstable if *x* prefers *y* to its assigned college, and meanwhile *y* prefers *x* to one of its admitted students. The unstable pair indicates a way to improve the matching, such as assigning *x* to *y* in the instance. An assignment with no unstable pairs is called a stable assignment.

Moreover, in order to handle the possible weak ranking orders in stable matching, Erdil and Ergin design a polynomial-time stable improvement cycles algorithm for the computation of a student-optimal school choice when some ranking tie exists by discovering and removing the stable improvement cycles [29].

When formulating the content distribution problem as a two-sided stable matching problem, we take the content C as the left vertices *L* and the storage cells of roadside units U as the right vertices *R*, and initialize the edge set *E* with ∅. The bipartite graph is BG=<L,R,E>. Since the bipartite graph matching is one-to-one mapping, for those contents having more than one replica carried by different roadside units, a preliminary algorithm is required, which will be discussed later. Then, set the ranking rules to calculate the left vertices’ preference profile and the right vertices’ priority rankings. Thereafter, solve the stable matching problem using Gale–Shapley algorithm [30] and the stable improvement cycles algorithm.

We explain modelling the preference ranking of content and the priority ranking of a storage cell below. In order to achieve a high access ratio and a short access latency, a compromise solution is designed, in which the preferences and the priorities are calculated based on the corresponding prior knowledge.

A preference profile is a vector of linear orders as $P={\left(p_{k}\right)}_{k\in C}$, where $p_{k}$ denotes the preference ranking of content $c_{k}$ over all the storage cells. Take an arbitrary left vertex L(k), which represents content $c_{k}$, as an example. The preference of L(k) over an arbitrary right vertex R(j,z), which represents the *z*th storage cell in a roadside unit $u_{j}$, is denoted as $p(c_{k},u_{j})$. To calculate the preference, we set a response factor matrix as

(3) $$M=F^{T}\times G.$$

In $M={\left(m_{kj}\right)}_{k\in C,j\in U}$, $m_{kj}=\sum_{i\in V}f_{ik}g_{ij}$, which indicates the number of satisfied requests by a replica of $c_{k}$ carried by $u_{j}$. In this article, we set

(4) $$p\left(c_{k},u_{j}\right)=m_{kj}=\sum_{i\in V}f_{ik}g_{ij}.$$

Thus, the preference implies the access ratio of a content replica.

It is observed that the larger $p(c_{k},u_{j})$ is, the more requests will be satisfied because of the replica of $c_{k}$ at $u_{j}$. Therefore, in the data acquisition model, content $c_{k}$ prefers to be held by a roadside unit $u_{j}$ having a larger $p(c_{k},u_{j})$. To achieve this, sort the roadside units in a descending order of $p(c_{k},u_{j})$, which indicates the preference ranking of the left vertex L(k) over all the storage cells of roadside units. Especially noteworthy is that the different storage cells of a roadside unit have the same preference. Thus, the preference ranking is probabilistic to be in a weak order, which should be addressed via the stable improvement cycles algorithm.

In addition, a priority ranking is a vector of linear orders $Q={\left(q_{j}^{z}\right)}_{j\in U,z\in \{1,2,...,w(j)\}}$ where $q_{j}^{z}$ is the preference of a storage cell R(j,z) over all the contents C. Take a right vertex R(j,z) as an instance; denote the priority of R(j,z) over a left vertex L(k) as $q(u_{j},c_{k})$. Note that the different storage cells of a roadside unit have the same priority ranking. To compute the priority, an encountering latency factor matrix is formulated as

(5) $$N=H^{T}\times F.$$

In $N={\left(n_{jk}\right)}_{j\in U,k\in C}$, $n_{jk}=\sum_{i\in V}h_{ij}f_{ik}$, which implies the total number of passing units before the vehicles obtain $c_{k}$ from $u_{j}$. Here, we take the average number of passing units as the priority $q(u_{j},c_{k})$, indicating the average access latency relevant with the replica of $c_{k}$ at $u_{j}$. Thus,

(6) $$q\left(u_{j},c_{k}\right)=\left\{\begin{array}{ccc} \frac{1}{m_{kj}}\cdot \sum_{i\in V}h_{ij}f_{ik} & , & m_{kj}>0;\\ |U|+1 & , & m_{kj}=0. \end{array}\right.$$

We see that the smaller $q(u_{j},c_{k})$ is, the shorter latency $u_{j}$ has to reply to the requests of $c_{k}$. Hence, in the data acquisition model, a roadside unit $u_{j}$ gives priority to content $c_{k}$ with a smaller $q(u_{j},c_{k})$ to occupy its storage cells. Based on this analysis, sort the contents in an ascending order of $q(u_{j},c_{k})$, which reflects the priority ranking of the right vertex R(j,z) over all the contents.

In our model DAS, the preference profile of the contents indicates the access ratio, while the priority rankings of the storage cells imply the access latency. Using the Gale–Shapley algorithm, we compute a stable matching of the original bipartite graph, denoted by GS=<L,R,ES>. For each edge linking, a left vertex L(k) with a right vertex R(j,z) in ES, the control center allocates a replica of $c_{k}$ to the *z*th storage cell of $u_{j}$. In this way, a stable replication solution with a tradeoff between the access ratio and the access delay is returned. Note that we do not use *M* to calculate $q(u_{j},c_{k})$ and *N* to compute $p(c_{k},u_{j})$ in that (1) in the Gale–Shapley algorithm, the left vertices have priorities to select matching nodes compared with the right vertices; (2) DAS aims for a high access ratio first and then an access delay as short as possible.

**Figure 2:**
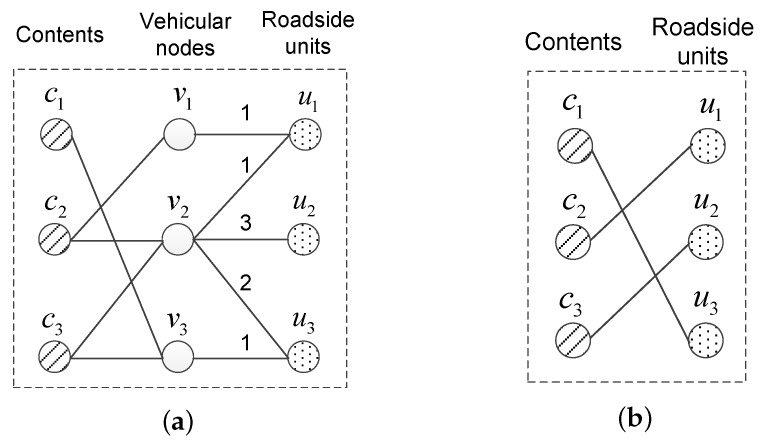
An instance of stable matching. (**a**) the relationships among C, V and U; (**b**) the stable matching.

An instance is given to clearly demonstrate the above process. In a vehicular network with three vehicular nodes $V=\{v_{1},v_{2},v_{3}\}$, three roadside units $U=\{u_{1},u_{2},u_{3}\}$ and three pieces of content $C=\{c_{1},c_{2},c_{3}\}$, each roadside unit has only one storage cell. Figure 2a displays the content request relationships between the contents and the vehicular nodes in τ and the encountering relationships between the vehicular nodes and the roadside units in τ+1. The weight of an edge is the index of a roadside unit in those units which will be met in sequence by a vehicular node. For example, the edge linking $c_{1}$ and $v_{3}$ means that $v_{3}$ requests $c_{1}$ in τ, and the weight 3 of the edge linking $v_{2}$ and $u_{2}$ means that $u_{2}$ is the third roadside unit, which $v_{2}$ will meet in τ+1.

In this instance, the preference profile of contents and the priority rankings of roadside units are listed in Table 2 and Table 3, e.g., $c_{1}$ is indifferent between $u_{1}$ and $u_{2}$ and prefers $u_{3}$ to both of them. Using the Gale–Shapley algorithm, a stable matching is shown in Figure 2b. Tie-breaking is unnecessary because there exists no stable improvement cycle. Therefore, the solution is that $c_{1}$, $c_{2}$ and $c_{3}$ are distributed to $u_{3}$, $u_{1}$ and $u_{2}$, respectively. In this way, the access ratio is 80% (the request of $c_{1}$ from $v_{3}$, the requests of $c_{2}$ from $v_{1}$ and $v_{2}$, and the request of $c_{3}$ from $v_{2}$ are completed, but the request of $c_{3}$ from $v_{3}$ cannot be completed), and the average number of passing units to access the contents is 1.5 (the numbers of passing units for the above four satisfied requests are 1, 1, 1, and 3 individually).

### 4.2. Multi-Replica Content Preprocessing Algorithm

In a vehicular network, a content may be replicated at more than one roadside unit, while the stable matching algorithm only supports one-to-one mapping. In order to address this contradiction, a multi-replica content preprocessing algorithm, as displayed in Algorithm 1, is conducted before executing the problem transformation.

**Algorithm 1:** Multi-replica content preprocessing algorithm.
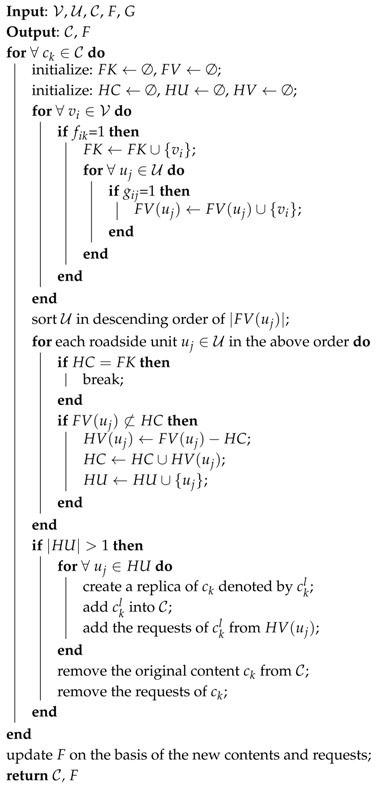


This algorithm aims to generate a minimum number of replicas for every potential multi-replica content, each of which is regarded as an independent content and serves for a part of the original requests in the following stable matching process. Five variables are introduced, including: FK is the vehicular nodes requesting $c_{k}$; $FV(u_{j})$ is the vehicular nodes which request $c_{k}$ and will meet $u_{j}$; HC is the vehicular nodes whose requests of $c_{k}$ could be satisfied already; HU is the roadside units which need to carry a replica of $c_{k}$; $HV(u_{j})$ is the vehicular nodes which need to download $c_{k}$ from $u_{j}$. In addition, the replication checking is carried out by U in descending order of $\left|FV(u_{j})\right|$. It helps to reduce the number of replicas to be generated because a roadside unit which may satisfy more requests should have a higher possibility to carry one replica, and hence the number of remaining unsatisfied requests decreases quickly. This algorithm substitutes several new independent contents each having one replica for content having two or more replicas. In this way, a one-to-many mapping is formulated as several one-to-one mappings, and hence the Gale–Shapley algorithm works.

**Figure 3:**
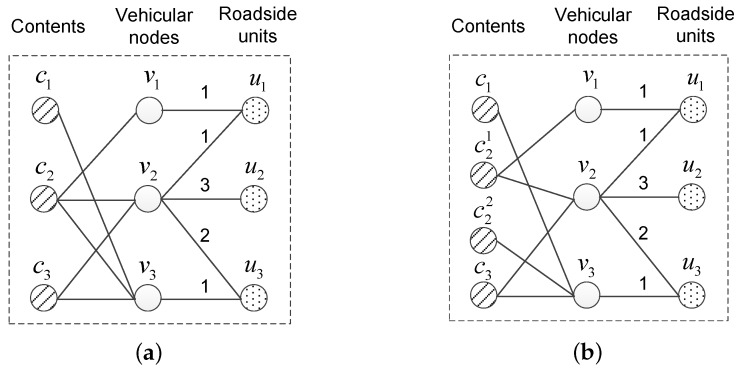
An instance of multi-replica content preprocessing. (**a**) original relationships; (**b**) after multi-replica content preprocessing.

Still discussing the instance shown in Figure 2, we assume $v_{3}$ also requests $c_{2}$. In other words, $c_{2}$ has three requests from $v_{1}$, $v_{2}$ and $v_{3}$, as displayed in Figure 3a. Considering the predicted encountering information, it is obvious that one replica of $c_{2}$ cannot serve all its requests. After multi-replica content preprocessing, as illustrated in Figure 3b, two new replicas denoted by $c_{2}^{1}$ and $c_{2}^{2}$ are created to replace the original $c_{2}$. $v_{1}$ and $v_{2}$ request $c_{2}^{1}$, while $v_{3}$ requests $c_{2}^{2}$. Thereafter, as two independent pieces of content in the stable matching, $c_{2}^{1}$ and $c_{2}^{2}$ will be allocated to two storage cells in different roadside units.

## 5. Data Acquisition via V2V Communications

After content distribution, when a vehicular node passes a roadside unit carrying the contents which are requested, it receives these contents from the roadside unit. However, when several vehicular nodes communicate with the same roadside unit simultaneously (which frequently happens in medium-high density vehicular networks), some may not obtain its required data due to bandwidth competition. Since the control center only distributes no more than one copy for each request at roadside units, the failed content delivery may have no chance to continue in the whole storage cycle. Therefore, how to help these vehicular nodes to acquire data becomes a challenging task.

Considering that a vehicular node could receive content from others via V2V communications, we regard vehicular nodes as the supplementary storage devices so as to increase the access ratio [31]. The supplementary storage starts in V2U communications when some vehicles request to and receive extra contents from roadside units. We call these vehicles the assistant vehicles and the extra contents as the assistant contents. After that, when the assistant vehicles encounter the vehicular nodes requesting the assistant contents, they reply to the requests through V2V communications.

### 5.1. Supplementary Storage at Vehicular Nodes

A principle is that the supplementary storage should not interfere with the regular content storage and transmissions. The assistant contents are stored in a particular space, namely, the assistant storage. The priority to occupy the storage space is given to the contents that the assistant vehicles themselves request. Beyond these, if they still have free storage cells, they could use them as the assistant storage. In addition, in V2U communications, the roadside units respond to the content requests first and then the supplementary storage requests.

In order to improve the supplementary storage efficiency, three problems need to be solved: (1) When a vehicle requests assistant contents, which contents should be sent by the roadside unit? (2) How long will the assistant vehicles carry the assistant contents? Or when will the assistant vehicles drop the assistant contents? (3) How will the assistant vehicles forward the assistant contents via V2V communications? Discussions about these issues are as follows.

**Figure 4:**
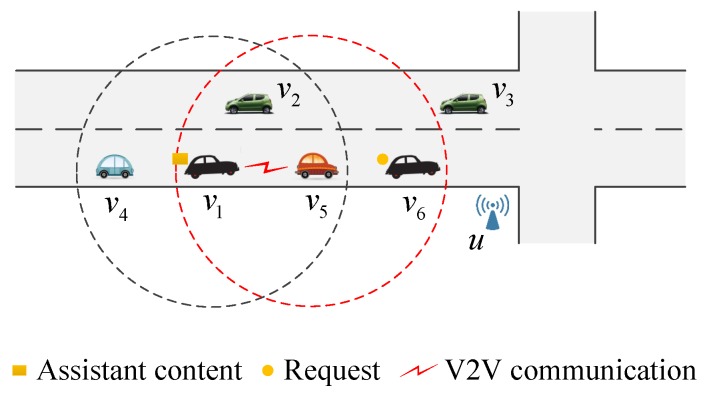
An instance of the forwarding of assistant contents.

(1) The selection of assistant contents. To increase the access ratio, the content selection depends on the transmission failures at roadside units and the routing plans of vehicular nodes. On the one hand, each roadside unit records its failed contents. When the transmission of a content $c_{k}$ from a roadside unit $u_{j}$ to a request sender $v_{i}$ fails due to bandwidth competition, $u_{j}$ finds the next roadside unit $v_{i}$ will meet, denoted by $u_{next}$, from g(i), and then increases the number of failed requests of $c_{k}$ related to the path from $u_{j}$ to $u_{next}$, namely, the failure frequency, denoted by $Fr(c_{k},u_{next})$ ($Fr(c_{k},u_{next})$ is initialized as 0 at the start of τ+1). On the other hand, a roadside unit selects assistant contents according to the failure frequency. When $v_{i}$ requests an assistant content from $u_{j}$, $u_{j}$ obtains $u_{next}$ from g(i) and sends a copy of the content having the biggest $Fr(c_{k},u_{next})$ to $v_{i}$.
  The selection of assistant contents gives priority to those having more unsatisfied requests, since there is a higher probability to meet the request senders. In addition, considering that vehicles only send data along its moving direction, different failure frequencies are calculated for different directions.
(2) The lifetime of assistant contents. After $v_{i}$ receives an assistant content $c_{k}$ from $u_{j}$, $v_{i}$ moves from $u_{j}$ to $u_{next}$ carrying $c_{k}$ and sends it to other vehicles requesting $c_{k}$. The supplementary storage process ends with dropping $c_{k}$ when $v_{i}$ reaches $u_{next}$.
(3) The forwarding of assistant contents. In order to accelerate the transmission of assistant contents, when an assistant vehicle encounters other vehicles, it selects the neighbor in the same moving direction and nearest to the next roadside unit and then forwards the assistant content to this neighbor. As Figure 4 shows, a vehicle $v_{1}$ has an assistant content and its next roadside unit to meet is *u*. In V2V communication, $v_{1}$ has three neighbors, i.e., $v_{2}$, $v_{4}$ and $v_{5}$. According to the above method, since $v_{5}$ is the neighbor nearest to and moving for *u*, $v_{1}$ forwards the assistant content to $v_{5}$. Furthermore, $v_{5}$ has a neighbor $v_{6}$ who requests this content, so $v_{5}$ replies to $v_{6}$. It is apparent that the data forwarding helps to shorten the access latency.

### 5.2. Incentive Strategy for Supplementary Storage

Although from the standpoint of the whole system, the supplementary storage improves the access ratio by exploring available storage capacity and communication opportunity, it indeed consumes the storage and communication resources of the assistant vehicles. Thus, vehicular nodes may not volunteer to participate in the supplementary storage. We put forward an incentive strategy to do with this conflict based on a metric, named the assistant score, which implies the contribution of a vehicular node in the supplementary storage. The assistant score of $v_{i}$ is denoted by S(i). The two core aspects of this strategy are discussed below.

(1) How to gain the assistant score. A vehicular node gains its assistant score when it takes part in the supplementary storage. Specifically, when a vehicular node $v_{i}$ obtains assistant content from a roadside unit and carries it for a carry-recording time $t_{Sc}$, $v_{i}$ gains a carrying score Sc and updates its assistant score as S(i)=S(i)+Sc; when $v_{i}$ forwards an assistant content to its neighbor or delivers it to its request sender, $v_{i}$ gets a transmitting score St and updates as S(i)=S(i)+St. In the simulation experiments, we set $t_{Sc}=10$ s, Sc=1, St=5.
(2) How to use the assistant score. As analyzed above, when several vehicles ask a roadside unit for different contents simultaneously, the roadside unit with limited bandwidth needs to send out these contents in some order. In our incentive strategy, this order is set based on the assistant scores of the request senders. In detail, the roadside unit computes the sum of the assistant scores of those vehicles requesting the same content, takes this value as the sending priority of the content, and sends these contents in descending order of their priorities until the communication chance passes by. For instance, four vehicular nodes, i.e., $v_{1}$, $v_{2}$, $v_{3}$ and $v_{4}$, send content requests to a roadside unit *u*. Their assistant scores are 5, 6, 4 and 8, respectively. $v_{1}$ and $v_{2}$ would like the content $c_{1}$, while $v_{3}$ and $v_{4}$ request $c_{2}$. We get the sending priority of $c_{1}$ is $S_{1}+S_{2}=11$ and that of $c_{2}$ is $S_{3}+S_{4}=12$. Since $c_{2}$ has a higher sending priority than $c_{1}$, *u* sends $c_{2}$ to $v_{3}$ and $v_{4}$ first. Then, if the communication chance still exists, *u* sends $c_{1}$ to $v_{1}$ and $v_{2}$.

To sum up, the supplementary storage at vehicular nodes makes better use of the storage space and the communication bandwidth, achieves more fair transmission scheduling at roadside units via the incentive strategy, and therefore further improves the access ratio in cooperative vehicle-infrastructure systems.

## 6. Performance Evaluation

### 6.1. Network Configurations

The performance evaluation of our data acquisition model DAS is conducted on a simulated vehicular network with the environment configurations shown in Table 4. According to IEEE 802.11p, the communication radius of vehicular nodes and roadside units is 250 m and the data rate is 5 Mbps. For the mobility model, after a vehicle sets a destination, it selects a random speed from a preset range and moves to its destination along the map-based shortest path [32] at this speed. Moreover, we will analyze the effects of several parameters, including the storage cycle, the size of assistant storage, the communication radius and the request model in Section 6.3.

In our previous research [33], we found that the data acquisition using roadside units had much better performances than those only using vehicular nodes because of the infrastructure with rich resources. Therefore, in our experiments, we compare those models using roadside units. In detail, we use a greedy replication algorithm as comparison, namely, Greedy. In Greedy, the control center prefers to distribute content a vehicle $v_{i}$ requests to the first roadside unit $v_{i}$ will meet (the roadside unit $u_{j}$ having $h_{ij}=1$). If the storage cells of this unit are full, then the content is distributed to the next roadside unit ($h_{ij}=2$) and so forth. It is apparent that Greedy aims for a short access latency firstly and a high access ratio secondly. In addition, in order to see the influences of the preference model and the priority model, a variant of DAS named vDAS is implemented, which takes the encountering latency factor as the preference of content over storage cells, and takes the response factor as the priority of a storage cell over contents. In addition, another compared method only has the content distribution at roadside units, which is generated by removing the supplementary storage from DAS, denoted by DAS-RU, so as to show the specific benefits of V2U or V2V communications.

The simulation experiments evaluate four criteria, i.e., the access ratio, the average access delay, the number of content replicas, and the transmission overhead. The access ratio is the ratio of those satisfied requests to all the generated requests. A higher access ratio implies a better performance. The average access delay is calculated for those completed requests. An access delay of a request is the duration from the time this request is sent out to that it is replied to. A method with a short average access delay works well in those vehicular applications having a strict delay requirement. The number of content replicas directly indicates the storage cost and hence the storage efficiency, while the transmission overhead, which is the number of transmissions in the entire network, shows the consumption of communication resources.

### 6.2. Simulation Results

**Figure 5:**
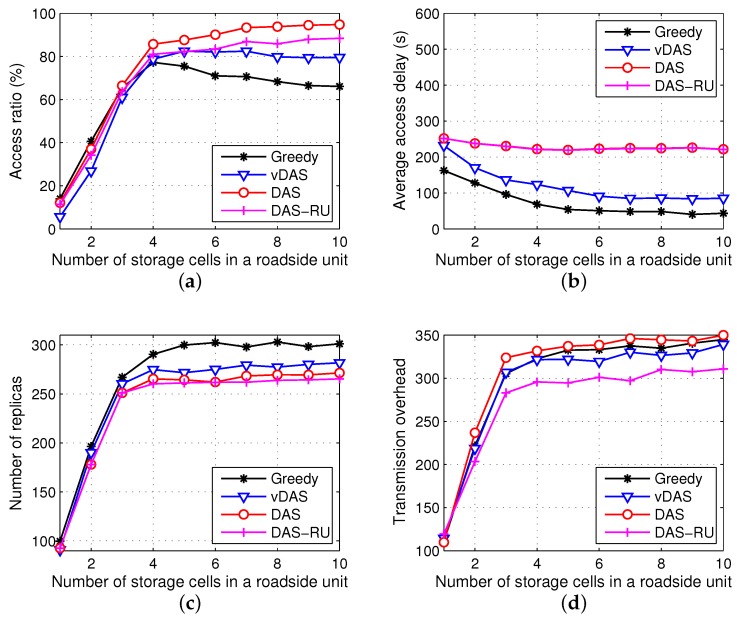
Simulation results. (**a**) access ratio; (**b**) average access delay; (**c**) number of content replicas; (**d**) transmission overhead.

In order to show the influence of the storage size, let the number of storage cells in a roadside unit (w(j), for ∀j∈U) range from 1 to 10. The simulation results of the four data acquisition models, i.e., Greedy, DAS, vDAS and DAS-RU, are illustrated in Figure 5. Since the contents are distributed according to the present network information in each storage cycle, the replication solutions in different cycles are independent. For a simple presentation of reliable results, the simulation results in all the cycles are averaged.

From Figure 5a, when the storage space is small, the access ratios of DAS and DAS-RU are almost the same, while that of vDAS is a little lower. The main reasons lie in two aspects: (1) in the Gale–Shapley algorithm, the preferences of left vertices (contents) play a more important role than the priorities of right vertices (storage cells); (2) since vDAS uses the encountering latency factor to compute the contents’ preferences, the benefits of the response factor (indicating the delivery ratio) wears off. In addition, when there are sufficient storage spaces, the access ratio of DAS increases to approximately 95%, while that of Greedy drops. This is because the stable matching algorithm improves the resource utilization. In addition, as the storage size increases, the ratio difference between DAS and DAS-RU becomes bigger because of the severe bandwidth competition around roadside units, which results in an obvious advantage of the supplementary storage at vehicular nodes. Overall, compared with others, DAS has a high and stable access ratio.

As Figure 5b illustrates, with an increasing number of storage cells, the average access delays of all the models keep stable after some drops. Since they all consider the delivery delay when distributing contents, enough storage helps to disseminate the contents to nearby roadside units and hence shorten the latency. While Greedy keeps the shortest latency, DAS has the longest one. Nevertheless, the delay of DAS is around 37% of the lifetime of contents (600 s), which is acceptable in most of the vehicular applications.

We observe that, in Figure 5c, all the methods have similar numbers of replicas, when each roadside unit has less than three storage cells. When the storage size rises, Greedy occupies more storage spaces than DAS and vDAS by nearly 10%. Since Greedy replicates contents from a local view, the storage spaces in the whole network are not efficiently utilized. Additionally, DAS has a few more replicas than DAS-RU because vehicles also carry content replicas for supplement storage, especially when the bandwidth competition at roadside units is severe. In one word, compared with Greedy and vDAS, DAS keeps a small storage consumption.

In Figure 5d, the transmission overheads of DAS, vDAS and Greedy are similar, while DAS-RU has a small overhead due to no V2V communications. Comparing Figure 5c,d, we see that the number of transmissions is larger than the number of replicas. Besides the transmission failures, the main reason is that one replica at a roadside unit or a vehicular node may serve for multiple requests during different V2V or V2U contacts.

In conclusion, when w(j)=10, compared with Greedy, DAS has a higher access ratio and less storage cost by approximately 30% and 10%, respectively, with a longer access delay (the delay is around 37% of the content lifetime, which is acceptable in most vehicular services). In DAS, the supplementary storage at vehicular nodes increases the access ratio by about 6%. In addition, DAS has a high access ratio, while its variant vDAS has a short access delay. This implies that our data acquisition framework demonstrates different properties via adjusting the models of the preference and the priority. This flexibility may enlarge the application scope of our proposal.

### 6.3. Parameter Analysis

We carry out extra simulations to see the impacts of the storage cycle, the size of assistant storage, the communication radiuses of vehicles and roadside units, and the request model on the overall performances. Here, we use the configurations in Table 4, and each roadside unit has 10 storage cells.

#### 6.3.1. Storage Cycle Analysis

**Figure 6:**
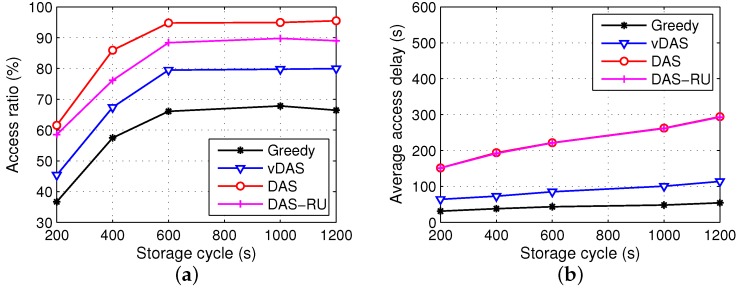
Results with different storage cycles. (**a**) access ratio; (**b**) average access delay.

Let the storage cycle be 200 s, 400 s, 600 s, 1000 s and 1200 s, respectively, and the numbers of storage cycles in the total simulation time 12,000 s are 60, 30, 20, 12 and 10, accordingly. The results are shown in Figure 6.

From Figure 6, we observe that when the storage cycle increases, the access ratios reach plateaus after sharp rises and the access delays always rise. On the one hand, the longer the storage cycle is, the more roadside unit candidates for the content carriers there are. Thus, the contents can be better allocated to roadside units with a result of higher access ratios. On the other hand, after the storage cycle reaches a threshold, an even longer cycle does little to help raise the access ratio since the bottleneck of the performance improvement changes to be the storage spaces of roadside units and the contact probability between vehicles and roadside units. Moreover, more elements in g(i) result in a longer delivery latency. As a conclusion, an appropriate storage cycle is important for DAS. In general, the storage cycle could be selected according to the network requirement of the transmission latency and by sampling analysis in pre-processing experiments.

#### 6.3.2. Assistant Storage Analysis

**Figure 7:**
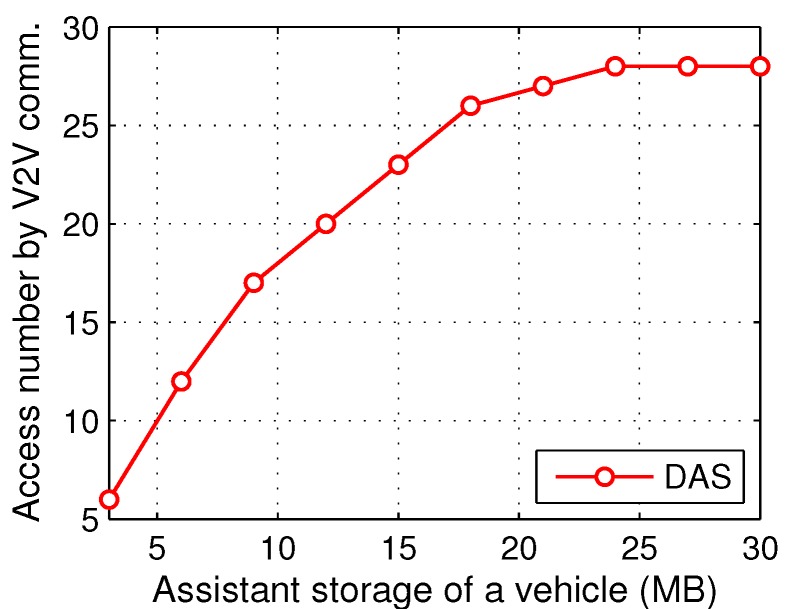
Results with different maximum sizes of assistant storage of a vehicular node.

The range of the maximum size of assistant storage in a vehicular node is from 3 MB to 30 MB, and the numbers of satisfied requests due to V2V communications in the whole simulation time are displayed in Figure 7.

Figure 7 shows that the larger the assistant storage is, the more contents are delivered because of the supplementary storage through V2V communications. However, after the size of assistant storage reaches 24 MB, the performance remains stable. This is because the delivery is still affected by the mobility model of vehicles, the bandwidth competition at roadside units and other factors. Searching for this turning point through some pilot study is helpful to achieve a full use of the assistant storage at vehicular nodes.

#### 6.3.3. Communication Range Analysis

The communication radius 250 we used above is in line with Dedicated Short-Range Communications (DSRC) standards, which is a specific standard for vehicle-to-vehicle communications, and some existing research also uses this value in their experiments, such as [34,35].

In urban scenarios, even though the safe distance between cars is affected by the velocity, 50 m is a conservative value when the velocity is lower than 100 km/h. Therefore, with transmission radius 250 m, in one lane, there might be 10 vehicles in the communication range. Taking Beijing commercial center as an example, since the roads usually have 4–6 lanes, there may be more communicating vehicles, especially in the rush hours. Although the control and scheduling of several contacts is challenging, integrating our scheme with scheduling methods like [36] is a potential solution in the future.

**Figure 8:**
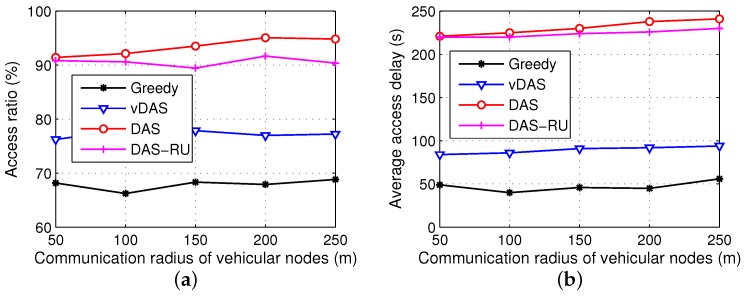
Results with different communication radiuses of vehicular nodes. (**a**) access ratio; (**b**) average access delay.

**Figure 9:**
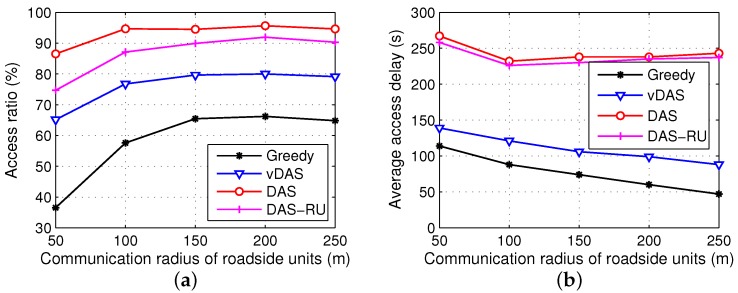
Results with different communication radiuses of roadside units. (**a**) access ratio; (**b**) average access delay.

In order to further analyze the effects of communication radius, we conduct two groups of simulation experiments with the communication radiuses of vehicular nodes and roadside units ranging from 50 m to 250 m, respectively. The results are displayed in Figure 8 and Figure 9.

As shown in Figure 8, the overall performances of all the schemes differ a little with different radiuses of vehicular nodes because a majority of the requests are satisfied by the roadside units rather than the inter-vehicle communications. However, we see that the difference between DAS and DAS-RU increases when the radius rises because of more encounters between vehicles.

From Figure 9, when the roadside units have larger communication ranges, the delivery ratios of all the schemes reach plateaus after some growths. Meanwhile, the access latencies of DAS and DAS-RU drop a little and then remain stable, while Greedy and vDAS have shorter delays. A large communication radius of roadside units is helpful to communicate with more vehicles, and deliver the contents early. In addition, with a small transmission range, more roadside units require the V2V cooperation to forward data, and hence DAS and DAS-RU have a larger difference on their performances.

#### 6.3.4. Request Model Analysis

**Figure 10:**
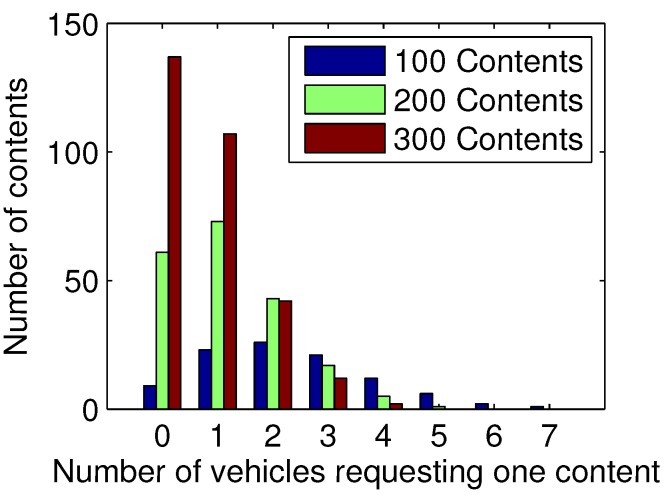
Popularity distribution of contents.

**Figure 11:**
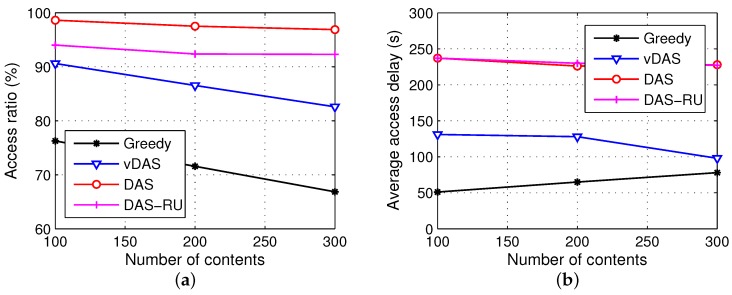

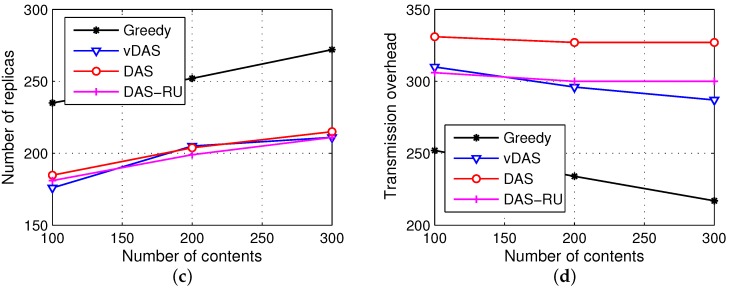
Results with different numbers of contents using Poisson distribution. (**a**) access ratio; (**b**) average access delay; (**c**) number of replicas; (**d**) transmission overhead.

Considering that the random requests are rare in real life, in order to simulate more realistic data requests, a series of experiments based on Poisson distribution are developed. On the one hand, the number of requests from each vehicle follows a Poisson distribution with λ=2.5, and hence 100 vehicles totally have 235 requests. On the other hand, we set different numbers of contents as 100, 200, and 300, and the number of requests for each content follow the Poisson distributions with λ = 2.35, 1.175, and 0.783, respectively. If content has a large number of requests, it is regarded as popular content. The popularity distributions in the three scenarios with 100, 200 and 300 contents are shown in Figure 10, and the performances of the four schemes are illustrated in Figure 11.

In Figure 10, we see that, given the number of requests in the entire network, when there are 100 pieces of content, nearly 70% of them have more than one request, and the most popular content has seven requests. By contrast, when there are 300 pieces of content, nearly half of them do not have requests, and the majority of the remaining contents have only one request for each. Therefore, a variety of contents leads to a sparse popularity distribution.

From Figure 11, a larger number of contents degrade the access ratio and bring in more replicas. Compared with the results using random requests in Figure 5, our scheme DAS with the requests following the Poisson distribution has a drop on the number of replicas because a replica of popular content may respond to multiple requests.

In general, from the parameter analysis, we conclude that DAS has a stable advantage over compared schemes on the metrics of delivery ratio and storage efficiency.

### 6.4. Performance Evaluation Using Real Taxi Trajectories

In order to further evaluate our proposal in real vehicular scenarios, we carry out experiments based on real taxi trajectories of 4600 taxis in Sanya City, Hainan Province, China, from 9 a.m. to 10 a.m. on 15 November 2016. The open access dataset is provided by Ministry of Transport of China [37]. The GPS data collection cycle is 10 s, but some vehicles have only one location entry in a minute.

**Figure 12:**
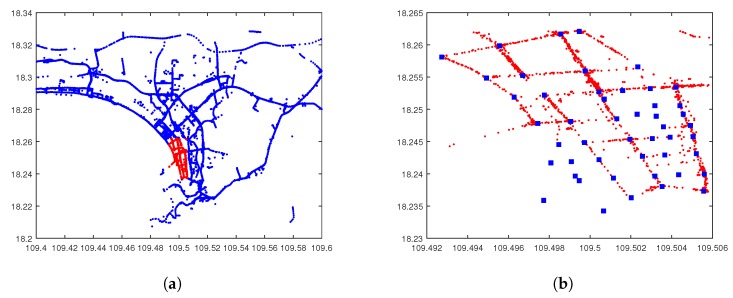
Real vehicular scenario. (**a**) Sanya City (China); (**b**) selected area.

To simplify the analysis, we select a square area in the center of the city with longitude in [109.493, 109.506] and latitude in [18.237, 18.262], as shown in Figure 12a, and the time period is from 9:00:00 a.m. to 9:01:59 a.m. (the storage cycle is 2 min). In this scenario, after deleting incorrect trajectories, there are 259 vehicles traveling on the selected roads. For some missing GPS data, we insert estimated locations according to the existing prior and next entries as well as the velocities. Additionally, we set 48 roadside units, almost all of which are at intersections. The map of our selected area with roadside units is illustrated in Figure 12b.

**Figure 13:**
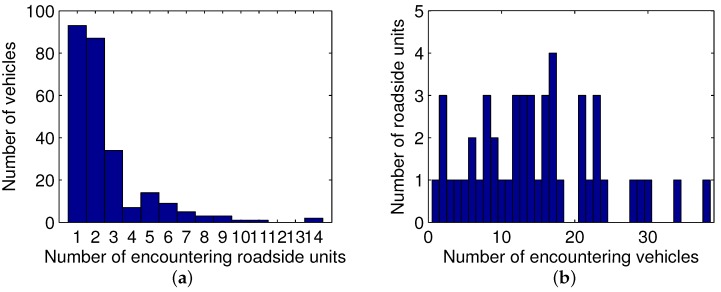
Encountering analysis. (**a**) distribution of the number of vehicles; (**b**) distribution of the number of roadside units.

Since the encountering of vehicles and roadside units significantly affects the overall performances, we get statistics about the number of contacts. The distribution of the number of vehicles, which will encounter a specific number of roadside units, is shown in Figure 13a, while the distribution of the number of roadside units, which will meet a specific number of vehicles, is displayed in Figure 13b.

As illustrated in Figure 13a, most of the vehicles encounter one or two roadside units during the two minutes, while two vehicles encounter 14 roadside units, which is the largest number of passing units. When a vehicle encounters more roadside units, the data it requested can be satisfied by any of these units, and hence the distribution solution has more flexibility to balance the requests from this vehicle and other vehicles in the network. Additionally, in Figure 13b, the majority of roadside units encounter less than 20 vehicles, while two units meet over 30 vehicles during the storage cycle. If a roadside unit has several encountering vehicles, its limited storage and bandwidth may be not enough to satisfy all of the requests from these vehicles. Therefore, an efficient distribution solution is highly required to improve the utilization of resources.

**Figure 14:**
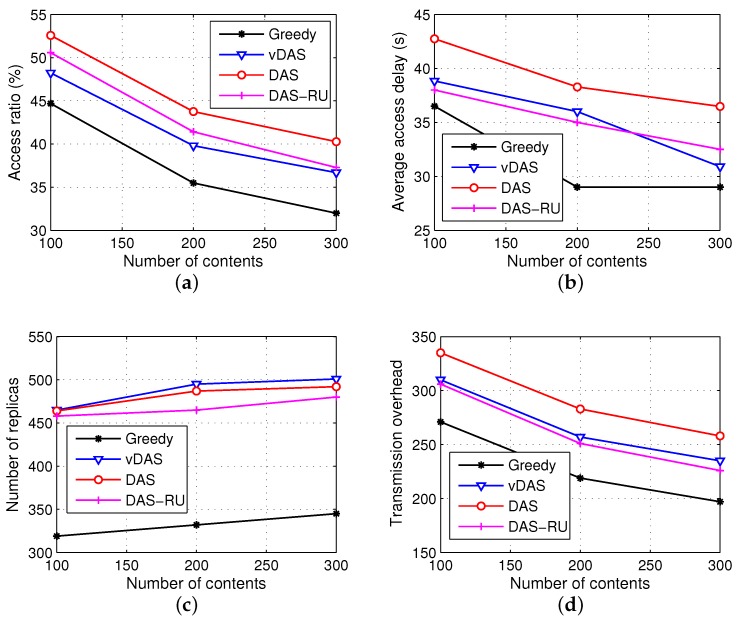
Results in real vehicular scenario. (**a**) access ratio; (**b**) average access delay; (**c**) number of replicas; (**d**) transmission overhead.

In the real scenario experiments, the parameter configurations are the same as the simulated experiments. Data requests follow the Poisson distribution, and let the number of contents be 100, 200 and 300, respectively. The performances of the four schemes are shown in Figure 14. We see that our scheme DAS has the highest access ratios, although they are lower than those in simulated scenarios, as a result of the variety of mobility. Note that the number of replicas in Greedy is the smallest because most vehicles only meet one or two roadside units and the Greedy scheme results in no data distributed for some vehicles in these few roadside units. In addition, the latency of DAS is shorter than 45 s, which is acceptable compared with the storage cycle 120 s. Overall, DAS has a high delivery ratio with an acceptable delay.

## 7. Conclusions

We propose a data acquisition model using stable matching of bipartite graph in cooperative vehicle-infrastructure systems, namely DAS, aiming for combining the two criteria, i.e., the access ratio and the access delay, in vehicular applications. In DAS, a bipartite graph with the contents as the left vertices and the storage cells of roadside units as the right vertices is constructed. Furthermore, the preference of content and the priority of a storage cell are modeled based on the response factor and the encountering latency factor, respectively, and the rankings on two sides are obtained. In this way, the data distribution problem is formulated as a stable matching problem, which is solved in polynomial time by the Gale–Shapley algorithm together with the stable improvement cycles algorithm. In order to deal with the contents having more than one replica, a multi-replica content preprocessing algorithm is designed to pave the way for one-to-one mapping in stable matching. In addition, vehicles execute the supplementary storage so as to satisfy those failed requests due to bandwidth competition at roadside units via V2V communications. Moreover, an incentive strategy is designed to motivate vehicles to carry and forward assistant contents. Finally, the experiments show that DAS has a high access ratio and a small storage consumption with an acceptable access latency.

However, there is still much profound research to be done to enhance the performance of our proposal. In simple simulated scenarios, the lower bounds of the metrics in theory could be helpful. Additionally, since the wireless channel at a roadside unit is a bottleneck, a solution to the traffic scheduling problem on the radio segment will further enhance the overall performance [38]. In addition, considering the cooperative downloading improves the information acquisition through inter-vehicle data transfers, the integration of our model with a cooperative downloading algorithm [39] has bright prospects.

## Figures and Tables

**Table 1:** Symbols.

Symbol	Description
V	Vehicular nodes, $V=\{v_{1},v_{2},...,v_{i},...\}$.
U	Roadside units, $U=\{u_{1},u_{2},...,u_{j},...\}$.
C	Contents, $C=\{c_{1},c_{2},...,c_{k},...\}$.
*V*	Index set of vehicular nodes, V={1,2,...,|V|}.
*U*	Index set of roadside units, U={1,2,...,|U|}.
*C*	Index set of contents, C={1,2,...,|C|}.
*s*	The size of content or a storage cell.
w(j)	The number of storage cells in $u_{j}$.
*F*	Content request matrix, $F={\left(f_{ik}\right)}_{i\in V,k\in C}$.
*G*	Encountering matrix, $G={\left(g_{ij}\right)}_{i\in V,j\in U}$.
*H*	Encountering latency matrix, $H={\left(h_{ij}\right)}_{i\in V,j\in U}$.
*P*	Preference profile of contents, $P={\left(p_{k}\right)}_{k\in C}$.
*Q*	Priority ranking of storage cells, $Q={\left(q_{j}^{z}\right)}_{j\in U,z\in \{1,2,...,w(j)\}}$.
τ	Current storage cycle.
τ+1	The next storage cycle.
$c_{k}^{l}$	The *l*th replica of a content $c_{k}$.
$Fr(c_{k},u_{next})$	Failure frequency of $c_{k}$ relevant with the path to $u_{next}$.
S(i)	The assistant score of $v_{i}$.

**Table 2:** The preference profile of contents.

$p(c_{k},u_{j})$	$u_{1}$	$u_{2}$	$u_{3}$	Ranking
$c_{1}$	0	0	1	$u_{3}≻u_{1}=u_{2}$
$c_{2}$	2	1	1	$u_{1}≻u_{2}=u_{3}$
$c_{3}$	1	1	2	$u_{3}≻u_{1}=u_{2}$

**Table 3:** The priority rankings of roadside units.

$q(u_{j},c_{k})$	$c_{1}$	$c_{2}$	$c_{3}$	Ranking
$u_{1}$	4	1	1	$c_{2}=c_{3}≻c_{1}$
$u_{2}$	4	3	3	$c_{2}=c_{3}≻c_{1}$
$u_{3}$	1	2	1.5	$c_{1}≻c_{3}≻c_{2}$

**Table 4:** Simulation environment configurations.

Parameter	Value
Network area	Grid network 7000×5000 m${}^{2}$, each grid 700×500 m${}^{2}$
Number of vehicles	100
Number of roadside units	10×10, deployed at the intersections
Number of contents	300
Simulation time	12,000 s
Storage cycle	600 s
Communication radius	250 m
Data rate	5 Mbps
Mobility model	Map-based shortest path model [32]
Mobility velocity	5.5∼16.5 m/s
Size of a content	3 MB
Number of requests	A vehicle sends at most 5 requests in τ
Contents requested	Randomly selected
